# Influence of the Lift-Off Effect on the Cut-Off Frequency of the EMAT-Generated Rayleigh Wave Signal

**DOI:** 10.3390/s141019687

**Published:** 2014-10-22

**Authors:** Pengxing Yi, Kang Zhang, Yahui Li, Xuming Zhang

**Affiliations:** 1 School of Mechanical Science & Engineering, Huazhong University of Science & Technology, Wuhan 430074, China; E-Mails: zhangkang@hust.edu.cn (K.Z.); liyh20@163.com (Y.L.); 2 School of Life Science & Technology, Huazhong University of Science & Technology, Wuhan 430074, China; E-Mail: zxmboshi@hust.edu.cn

**Keywords:** EMAT, cut-off frequency, rayleigh wave, lift-off

## Abstract

The electromagnetic acoustic transducer (EMAT), a non-contact NDT tool with large lift-off, is becoming an attractive method for detecting the cracks in the metal parts. However, the lift-off of the transducer has a direct effect on the feature that is used to characterize the defects. A detailed investigation on the relationship between the feature and the lift-off of the EMAT is crucial in the detection process. This paper investigates the lift-off effect on the feature, cut-off frequency of EMAT in the Rayleigh wave. The study can be divided into two parts. Firstly, with a multi-field coupling environment, 2-D electromagnetic and wave generation EMAT models are built to simulate the interaction of the Rayleigh wave with the surface crack. Then, the lift-off effect on the cut-off frequency is investigated through simulation and experiment. Compared to the previous studies, it is found that lift-off would cause a negative result when the lift-off varies in the testing process. Besides, the calibration obtained from the tests at a random lift-off value can be used in other tests with any different lift off value provided that the lift-off is kept as a constant during the detection process.

## 1. Introduction

In the remanufacturing process of construction machinery, metallic parts such as steel parts and aluminum parts are widely used. However, various defects will emerge on these metallic parts after being produced and utilized for some time. These defects will result in the deterioration of their functions. Non-destructive testing (NDT) is becoming a useful and essential tool in the inspection of these defects. A NDT technique should be able to inspect the metallic parts efficiently, accurately and timely. The conventional piezoelectric ultrasonic testing is a method that has been widely analyzed and used in non-destructive testing. Compared with the conventional piezoelectric transducer [1], the electromagnetic acoustic transducer (EMAT) is becoming a more attractive alternative. With a combination of static and dynamic magnetic field, the EMAT can transform the electromagnetic energy into acoustic energy [2]. The EMAT requires no coupling medium and can operate with a high speed or elevated temperature [3]. Besides, the EMAT can generate a variety of wave-modes more easily. However, the signal-to-noise ratio of EMAT is relatively low and the EMAT is sensitive to the variation of the lift-off [4–7]

In order to evaluate the defects in the metallic parts accurately, many features, such as the ultrasonic time of flight (TOF) [7,8], the transmission coefficient, the reflection coefficient [9,10] and so on, are studied. R.S. Edwards [11,12] used a low frequency and wideband signal to generate Rayleigh wave which is used to inspect the defects and put forward a new feature “cut-off frequency” to evaluate the defects. The feature is different from the cut-off frequency of the guided wave. For Rayleigh waves, the majority of the energy lies within the depth of one wavelength. When the depth of the crack is longer than the wavelength, the waves will be effectively blocked and the signal will be so small that it cannot be detected [13]. R.S. Edwards [11,12] made an accurate calibration and defined cut-off frequency corresponding to the wavelengths of less than 73% of the crack depth that was effectively blocked. The calibration is relevant to many parameters such as the coil turns, width and length of the conductors, as well as the lift-off distance. In addition, the parameters of the magnet, such as its dimensions and the magnetic field, may also affect the calibration. Among the parameters, the lift off between the probe and sample is crucial in the detection process. Based on the analysis in the linear coil [11] and meander coil [12], it is considered that the lift-off had little influence on accurate inspection of the feature. However, an in-depth study on the lift-off effect was not conducted. In this paper, we focus on studying the lift-off effect on calibration.

To understand the lift-off effect better, a finite element method is used in this paper. In the transmitting and receiving process, the electromagnetic energy and acoustic energy can transform into each other. The analysis of the interaction is a multi-field coupling problem. Many recent studies made use of software tools to analyze the multi-field coupling [14–18]. In this paper, a 2-D electromagnetic and wave generation model of EMAT is built to understand how the lift-off variables affect the cut-off frequency in the Rayleigh Wave through multi-physics field simulation with COMSOL, and the simulation results are verified in the experiment. Based on the simulation and the experimental results in this paper, it was found that the calibration of any lift-off value can be applied to others if lift-off is constant in the detection process. However, the feature “cut-off frequency” is sensitive to the variation of lift-off.

The rest of this paper is organized as follows. The principle of the establishment of the EMAT model is described in Section 2. In Section 3, the 2-D electromagnetic and wave generation model is built. In Section 4, we analyze the lift-off effect on the feature “cut-off frequency” of the EMAT in detail. The conclusion is given in Section 5.

## 2. Principle of EMAT

In the transmitting process, the EMAT transforms the electromagnetic energy into acoustic energy. The electromagnetic acoustic transducer consists of a coil which provides a dynamic current, a sample for testing and a magnet providing a static magnetic field. In the nonmagnetic material, the ultrasonic wave is generated by the Lorentz forces. While in the ferromagnetic material, the Lorentz forces, the magnetization forces and the magnetostrictive forces all have an impact on the generation of the ultrasonic wave [19,20]. In this paper, we conduct research on the non-ferromagnetic material.

**Figure 1.**
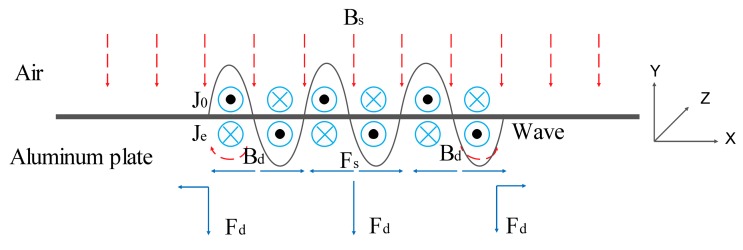
The Lorentz mechanism in the aluminum plate.

As shown in the Figure 1, the Rayleigh wave is generated by the Lorentz forces due to the interaction of the magnetic flux density and the eddy current induced in the aluminum plate. The magnetic flux density *B* contains the static magnetic flux density *B_s_* provided by the permanent magnet and the dynamic magnetic flux density *B_d_* induced by the alternating current in the meander coil. The dynamic Lorentz forces *F_d_* and static Lorentz forces *F_s_* interact constructively and destructively.

Meanwhile, in the receiving process, the EMAT transforms the acoustic energy into the electromagnetic energy. The receiving EMAT is a velocity sensor and can be used to measure the in-plane and out-plane velocity [10,21,22]. The relationship between the received signal and the velocity of the plate is shown as Equation (1) [21]. The received signal is proportional to the velocity of the plate. So, the variation of the normalised FFT received signal can be represented by the analysis of the normalised FFT velocity in the plate.

(1) $$V_{EMAT}=-2N\frac{1}{1-j{k_{c}}^{2}{\delta_{2}}^{2}/2}l_{0}•(v_{0}\times B_{0})$$

Therefore, in this paper , we pay attention to the buliding of transmitting process. In the transmitting process, the electromagnetic model shoud be bulit to calculate the force in the plate, and the wave generation model should be bulit to analyze the propogation of the wave.

By defining a vector magnetic potential A and considering the skin and the proximity effects [23–25], the coupling of the electromagnetic field and the elastic dynamic field is given by the equations as follows

(2) B=∇×A

In the coil domain

(3) $$\nabla \times (\frac{1}{\mu }\nabla \times A)=-\sigma \frac{\partial A}{\partial t}+J_{0}$$

In the air domain

(4) $$\nabla \times (\frac{1}{\mu }\nabla \times A)=-\varepsilon \frac{\partial^{2}A}{\partial t^{2}}$$

In the sample domain

(5) $$\nabla \times (\frac{1}{\mu }\nabla \times A)=J_{e}$$

(6) $$J_{e}=-\sigma \frac{\partial A}{\partial t}+\sigma \frac{\partial u}{\partial t}\times (\frac{1}{\mu }\nabla \times A)$$

(7) $$f=f_{s}+f_{d}$$

(8) $$f_{s}=J_{e}\times B_{s}$$

(9) $$f_{d}=J_{e}\times B_{d}$$

(10) $$\rho \frac{\partial^{2}u}{\partial t^{2}}=\nabla •T+f$$

where μ is the permeability and ε is the permittivity, *B* is the magnetic flux intensity, *J* is current density, σ is the electrical conductivity, ρ is the mass density, *u* is the elastic deformation, *f* is the force per volume, and *T* is the elastic stress tensor.

## 3. Model of the EMAT

### 3.1. Electromagnetic Model

**Figure 2.**
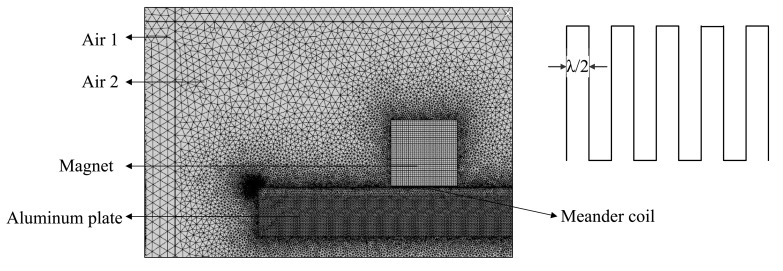
The schematic of the 2-D model in the COMSOL.

According to Equations (2)–(9), we calculate the force distribution in the aluminum plate. In this study, the *z* direction of the wire dimension is assumed to be larger than that in other two directions. Therefore, we can analyze the process in 2-D model ignoring the effect of the *z* direction. With different configurations of the magnet, coil and sample, there will be different force distributions and wave modes. A tone-burst pulse signal is sent through the meander coil consisting of six conductor wires. The peak to peak amplitude of the current is 50A, the frequency is 500 KHz and the periodicity is 3. The coil of the *x*-direction width is 0.165 mm, the *y*-direction height is 0.03 mm and *z*-direction length is 45 mm. At room temperature and under no stress conditions, the Rayleigh wave velocity at the 6061 aluminum plate is about 2930 m/s, so the wavelength is about 5.86 mm, and the interval between the adjacent coils is about 2.93 mm. The size of the permanent magnet is 40 × 40 mm and the remnant magnetic flux intensity is 1.21 T. Figure 2 is the schematic of the 2-D electromagnetic model.

In order to get the accurate electromagnetic field distribution and reduce the calculation time, the infinite element is used in the Air 1. With the use of the boundary layer on the surface of the plate, the skin depth has about eight elements. The magnet and coil are mapped into quadrilateral elements. The plate is divided into triangular elements. The air area 1 and air area 2 are divided into triangular elements with extremely fine mesh freely.

### 3.2. Wave Generation Model

**Figure 3.**
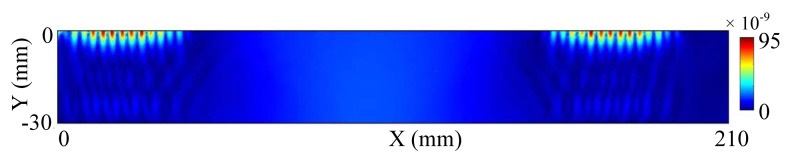
The propagation of the Rayleigh wave.

The force calculated in the electromagnetic model is the driving source in the wave generation model which is built in the solid mechanics module in COMSOL. According to Equation (10), we calculate the displacement distribution in the aluminum plate. The thickness of the plate is 30 mm, about 4∼5 times of the wavelength of the signal. For Rayleigh wave, when the depth is a few times of the wavelength, the strength of wave is so small that we can ignore it [10]. So, it is reasonable to set the boundary at the bottom of the plate as the low reflecting boundary. The wave propagates in the plate with free boundary in other three boundaries. The displacement of the surface wave is shown in Figure 3. It can be seen that the wave propagates in the two opposite directions.

**Figure 4.**
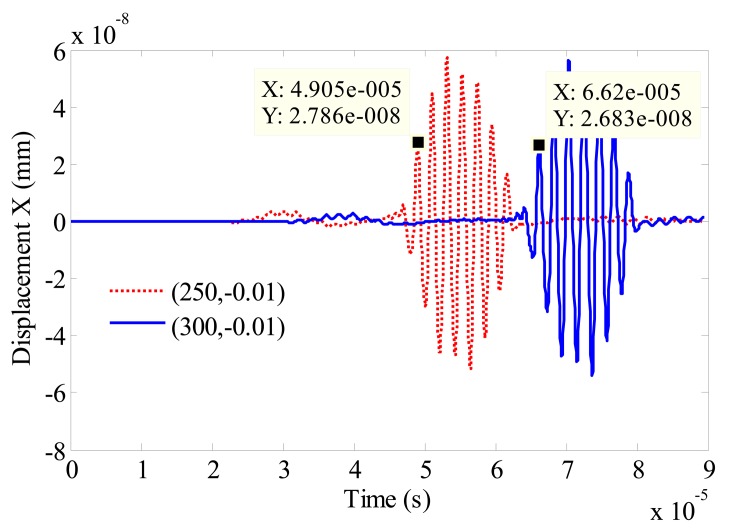
The in-plane displacement at position (300, −0.01) and position (250, −0.01).

The majority of the Rayleigh wave energy is concentrated within one wavelength below the surface of the plate. From Figure 3, the longitudinal length of the bright color region is about 6 mm which is in accord with the wavelength of signal. Figure 4 illustrates the in-plane displacement at position (300, −0.01) and position (250, −0.01). The arriving time of the first peak is 4.905e−5 s at position (250, −0.01), while the time is 6.62e−5 s at position (300, −0.01). The group velocity can be calculated as follows:

(11) $$\text{The Group Velocity}=\frac{\text{Distance}(\text{m})}{\text{Time of Flight}(\text{TOF}(\text{s}))}$$

where the time of flight between the two positions is (6.62e−5–4.905e−5) = 1.715e−5 (s), and the velocity calculated with this model is (300–250) e−3/1.715e−5 = 2915.45 m/s. The relative error between the calculated value and the theoretical value is 0.5%.

**Figure 5.**
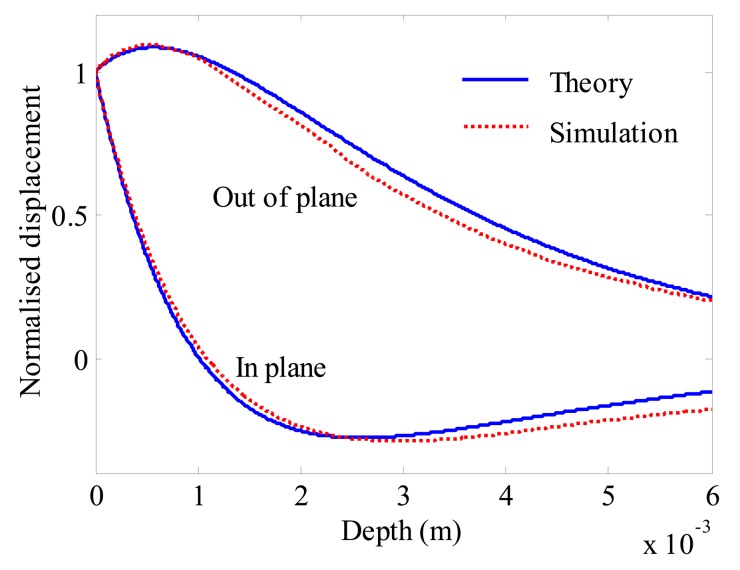
Normalised the out of plane and in plane displacement.

The out of plane and in plane displacement are normalised to each displacement on the surface of the sample. As shown in Figure 5, the differences of the in plane and out of plane displacement both are small. It is reasonable to prove that the simulation results are relatively accurate and the wave generated with this model is Rayleigh wave.

## 4. Experiments and Results

### 4.1. Experiment Details

**Figure 6.**
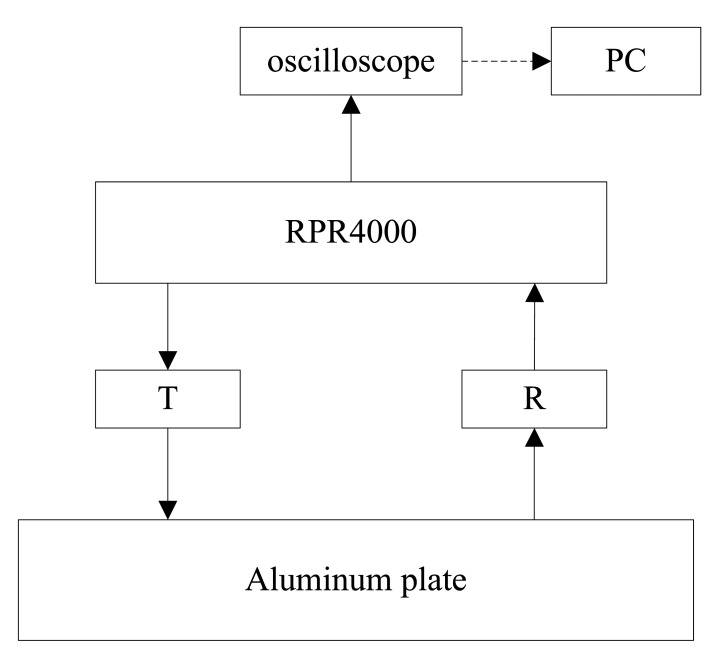
The schematic diagrams of experimental system.

As shown in Figure 6, a high power pulse generator and receiver RPR4000 is used to generate pulse current and receive the voltage induced in the receiving coil. The receiving signal is processed by RPR4000, then displayed and saved in the oscilloscope. Finally, the signal is analyzed by the PC.

**Figure 7.**
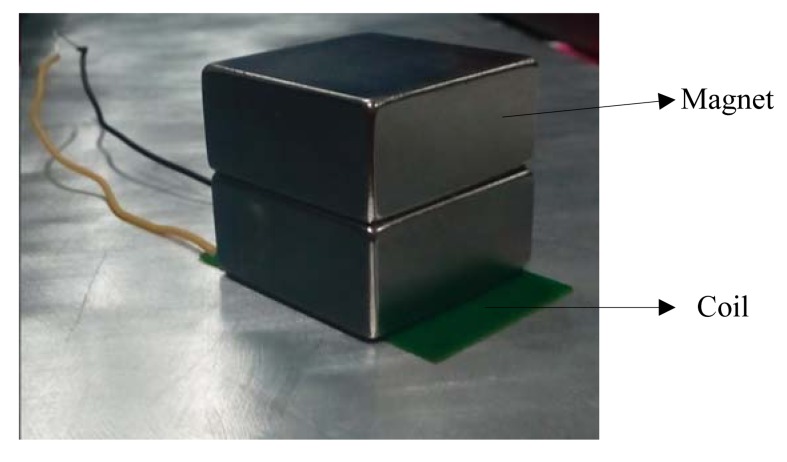
The excitating and receiving EMAT probe.

Figure 7 shows the structure of the excitating EMAT and the receiving EMAT probes which are the same. The coil is made of printed circuit board and the *z* direction length of the coil is 45 mm. The magent is made up of two magnets and each magnet’s dimension is 40 × 40 × 20 mm. The dimension of the plate used for experimental testing is 500 × 500 × 30 mm. The lift off is accomplished by a number of B5 papers inserted between the probe and the sample. The thickness of one B5 paper is almost 0.1 mm measured by micrometer. As shown in Table 1, the maximum relative error is 5%. The maximum lift off is 2.5 mm for a defect free plate, while for a plate with 2 mm defect, the maximum lift off is 1.4 mm.

**Figure 8.**
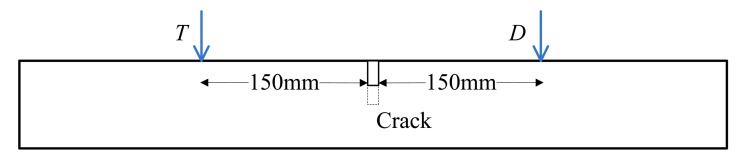
The transmitting, crack and detection position at the plate.

The transmitting position, the surface crack position and the detection position on the tested plate are simplified as Figure 8. The detection position is 150 mm away from the crack position to avoid the near-field enhancement [10].

### 4.2. Results and Discussion

In [11], R.S. Edwards analyzed the received signal, then applied a linear fit to the normalised FFT curves, and defined the frequency at which the linear fit crosses the frequency axis as the “cut-off frequency”, while in the deepest cracks, the frequency corresponding to the minimum value at the main region analyzed is used as the “cut-off frequency”.

**Figure 9.**
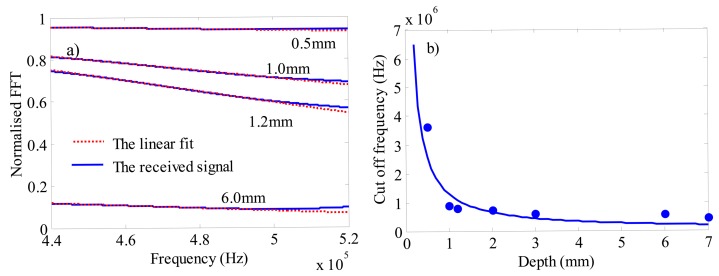
(**a**) The calculation of the cut-off frequency with simulation; (**b**) the relationship between the cut-off frequency and the depth of the crack with simulation.

Similar to R.S. Edwards’ study [11,12], we calculate the normalised FFT of the in plane velocity which is the integral over the penetration depth of the surface waves at detection position. Figure 9a depicts the normalised FFT of a signal with defect over signal without defect. It can be observed that the calculation process is a linear fit to the transmission coefficient at a certain range of the frequency. Figure 9b shows the relationship between the depth of the crack and the cut-off frequency, a calibration can be obtained based on the relationship illustrated in Figure 9b. A result similar to the experiment result in the former study [11,12] can be found in this simulation model. The bandwidth is narrower in meander coil than the one in linear coil, which will affect the accuracy. However, this divergence will not influence the analysis of the lift off effect as follows. The transmission coefficient will change if the parameters of probe differ. Therefore, a new calibration should be made for a different probe.

**Figure 10.**
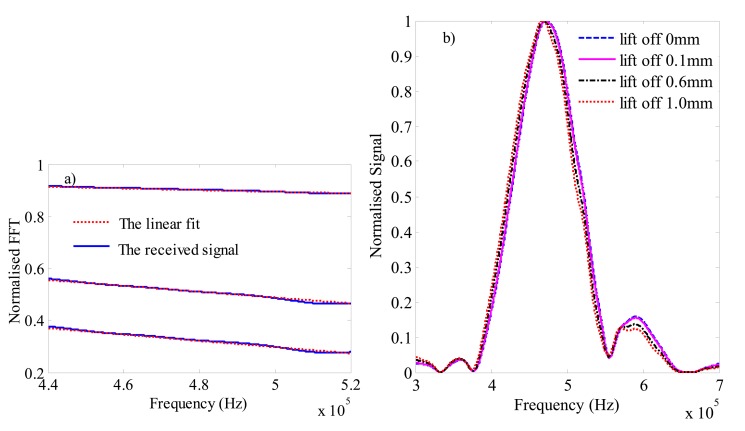
(**a**) The normalised simualtion results of the FFT at lift-off 0.1 mm, 0.6 mm and 1.0 mm to the FFT at 0 mm; (**b**) The normalised simualtion results of the FFT singals to the peak values at lift-off 0 mm, 0.1 mm, 0.6 mm and 1.0 mm, respectively.

By changing the lift-off from 0.1–1 mm, we calculate the normalised FFT in a defect free plate. Figure 10a shows the normalised simulation results of the FFT at different lift-off values over lift-off of 0 mm. As it is shown in Figure 10a, the normalised FFT signal is not parallel to the x axis and a finite cut-off frequency value is obtained. Figure 10b is the normalised simulation results of the FFT signals to the peak values at different lift-off values. Compared to the simulation results, the normalised FFT values of the experimental results are a little small. This difference is caused by a factor that the experimental results consider the out of plane velocity while the simulation does not. However, it does not make an impact on the analysis because our study mainly explores the tendency of the lift off impact on the cut-off frequency, rather than the quantitative results of the normalized FFT values.

**Figure 11.**
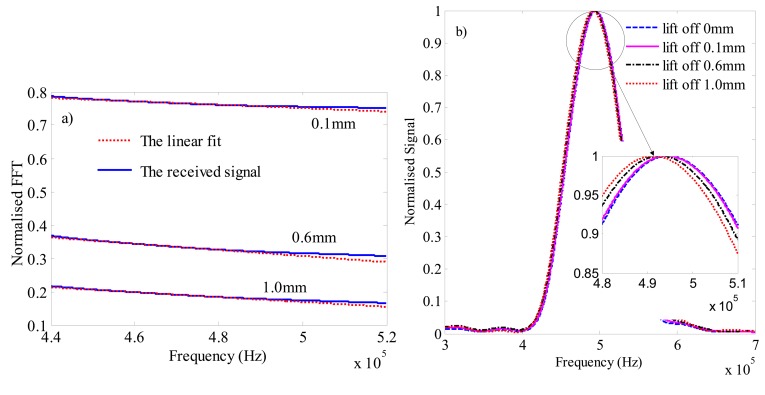
(**a**) The normalised experimental results of the FFT at lift-off 0.1 mm, 0.6 mm and 1.0 mm to the FFT at 0 mm; (**b**) The normalised experimental results of the FFT singals to the peak values at lift-off 0 mm, 0.1 mm, 0.6 mm and 1.0 mm, respectively.

Figures 10b and 11b show that the variation of the central frequency is small. As it is listed in Table 2, the maximal difference is 0.74%. Figure 10b illustrates that when the lift-off becomes larger, the Central-Frequency shifts to the low-frequency. The above phenomena are same with the previous study [11,12] and verified in the experiments shown in Figure 11b. However, this difference cannot be ignorable in the calculation of the cut-off frequency. The lift-off influence is not the same for different frequencies, the amplitude differences at the low frequency region are smaller than that at the high frequency region as shown in Figure 4b in the reference [12]. It means that the normalised FFT could not parallel the x axis and an infinite cut-off frequency cannot be calculated. If the lift-off varies, a finite frequency is obtained and applied in the calibration, which will signal a defect in a defect-free plate. Moreover, the cut-off frequency varies with different lift-off values. From Table 2, the maximal difference of the cut-off frequency is 46.9%.

**Figure 12.**
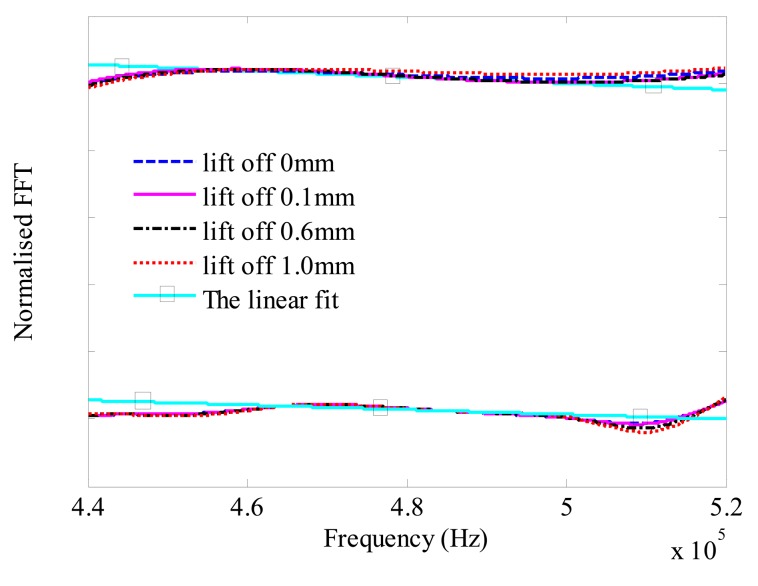
Normalised the FFT at 2 mm depth to the signal in the free defect plate at lift-offs of 0 mm, 0.1 mm, 0.6 mm and 1.0 mm.

If we keep the lift-off value unchanged in the detection process, we normalise the FFT signal at different depths to the FFT signal in the free plate. The simulation results and the experimental results at 2 mm depth are shown in Figure 12. In order to calculate the cut-off frequency, two linear fits are added to the experimental result and the simulation result at lift-off is 0 mm. The experimental results are offset by a distance to distinguish the simulation results. The transmission coefficient is less sensitive to the lift-off. Therefore, the FFT signals should be almost the same at different lift-offs. As shown in Figure 12, the normalised FFT at lift-off of 0 mm almost coincides with the FFTs at lift offs of 0.1 mm, 0.6 mm and 1.0 mm. In other words, the cut off frequencies calculated by the linear fit curves are almost the same. The difference of the cut-off frequency at lift-offs of 1.0 mm to 0 mm is slightly bigger than the difference at lift-offs of 0.1 mm to 0 mm through the calculation. However, compared to the variations of the frequency due to the crack, the differences are so small that they can be ignored. It means that the calibration obtained from the analysis of the lift-off value can be applied to other lift-off values.

## 5. Conclusions

The EMAT has been widely used in non-destructive testing nowadays because it does not require coupling medium and can operate at high temperatures and speeds. On the basis of the electromagnetic and elastic equations, a 2-D electromagnetic model and a wave generation model are developed in this study. The electromagnetic model is built to calculate the Lorentz force density which is the driving force density in the generation of the wave. It is reasonable to regard the generated wave in this model as a Rayleigh wave because the group velocity, energy of the wave and the normalised displacement are in accordance with the theoretical analysis.

By analyzing the change of the in-plane velocity with the depth of the crack, it is found that the relationship between the feature cut-off frequency and the depth of the crack analyzed in the meander coil is similar to that obtained in the linear coil [11] and the meander coil [11,12]. Furthermore, a calibration can be obtained from the relationship. The calibration obtained from the lift-off at 0 mm analyzed in this paper can be applied to the others if the lift-off is unchanged in the inspection process. This can reduce the inspection time. However, we will make a severe judgment error if the lift-off varies in the inspection process. Compared with the analysis based on the meander coil in [12], our work has provided a clear interpretation of the lift-off effect on the feature cut-off frequency.

Surely, the calibration may also be influenced by other parameters than the lift off. Besides, the lift-off is also influenced by other parameters of EMAT, such as the dimension of the magnet. In future study, much work should be done to provide an in-depth understanding of how other parameters influence the cut-off frequency of EMAT.

## Figures and Tables

**Table 1.** The lift off accomplished by the B5 paper.

**Lift off**	**0.1 mm**	**0.3 mm**	**0.6 mm**	**1.0 mm**	**1.5 mm**	**2.0 mm**
Number of papers	1	3	6	10	15	20
Measured value	0.095 mm	0.288 mm	0.590 mm	0.990 mm	1.483 mm	1.968 mm
Relative error	5.0%	4.0%	1.6%	1.0%	1.1%	1.6%

**Table 2.** The experimental results of the variation of the center frequency and cut-off frequency.

**Lift-off Value (mm)**	**Central Frequency (MHz)**	**Cut-off Frequency (MHz)**
0	0.495	---
0.1	0.4945	1.0054
0.6	0.493	0.7669
1.0	0.4913	0.5334
